# Passion, Trait Self-Control, and Wellbeing: Comparing Two Mediation Models Predicting Wellbeing

**DOI:** 10.3389/fpsyg.2017.00841

**Published:** 2017-05-29

**Authors:** Walid Briki

**Affiliations:** Sport Science Program, College of Arts and Sciences, Qatar UniversityDoha, Qatar

**Keywords:** personality, self-regulation, harmonious passion, obsessive passion, psychological health

## Abstract

Research has found that passion and trait self-control represented key determinants of wellbeing. Yet, no study to date has attempted to investigate the mediating influences of trait self-control and passion for accounting for the relationships between passion, trait self-control, and wellbeing (dependent variable). Using different frameworks, such as the dualistic model of passion and the neo-socioanalytic theory, the present study proposed two mediation models, considering either trait self-control (model 1) or passion (model 2) as the mediating variable. Five hundred nine volunteers from the United States (326 females and 183 males; *M*_age_ = 31.74, *SD*_age_ = 11.05, from 18 to 70 years old), who reported being passionate about a specific activity (e.g., fishing, swimming, blogging; *M*_passion_ = 5.94, *SD*_passion_ = 0.89), answered questionnaires assessing harmonious and obsessive passion, trait self-control, and wellbeing (measured through hedonic and eudaimonic wellbeing scales). Preliminary analyses revealed that both models were significant (*model 1:* absolute GoF = 0.366, relative GoF = 0.971, outer model GoF = 0.997, inner model GoF = 0.973, *R*^2^ = 18.300%, *p* < 0.001; *model 2:* absolute GoF = 0.298; relative GoF = 0.980; outer model GoF = 0.997; inner model GoF = 0.982; *R*^2^ = 12.111%, *p* < 0.001). Correlational analyses revealed positive relationships between harmonious passion, trait self-control, and wellbeing, and no relationships of obsessive passion with trait self-control and wellbeing. Mediation analyses revealed that trait self-control significantly mediated the relationship between harmonious passion and wellbeing (i.e., partial mediation, VAF = 33.136%). Harmonious passion appeared to significantly mediate the positive effect of trait self-control on wellbeing; however, the size of the mediating effect indicated that (almost) no mediation would take place (i.e., VAF = 11.144%). The present study is the first to examine the relationships between passion, trait self-control, and wellbeing, and supports the view that trait self-control and harmonious passion represent not only adaptive and powerful constructs, but also key determinants of wellbeing. Implications for the study of passion, trait self-control and wellbeing are discussed.

## Introduction

Positive psychology is “…the scientific study of what makes life worth living” (Peterson and Park, 2014, p. 2). In that regard, the questions raised by that field of study are about happiness and wellbeing. Wellbeing encompasses two forms: Hedonic (or subjective) and eudaimonic wellbeing. Hedonic wellbeing corresponds to “…people's evaluations of their lives—the degree to which their thoughtful appraisals and affective reactions indicate that their lives are desirable and proceeding well” (Diener et al., 2015, p. 234). Eudaimonic wellbeing is defined as “…the striving for perfection that represents the realization of one's true potential” (Ryff, 1995, p. 100). In other words, hedonic wellbeing emphasizes the pursuit of pleasurable experiences, whereas eudaimonic wellbeing emphasizes the pursuit of a meaningful life (Peterson and Park, 2014; Vallerand, 2015). Wellbeing has elicited the interest of politicians, economists, philosophers, and psychologists around the world since studies revealed that happiness predicted performance, moral behavior, health, and longevity (e.g., Diener, 2000; De Neve et al., 2013; Diener et al., 2015). Therefore, besides the fact that wellbeing represents a significant interest to a worldwide audience, examining its predictive factors is of great importance.

Vallerand (2015) argued that being “…passionate about a meaningful activity (or object or even a person) can provide joy and meaning to one's life that contributes to having a life worth living” (p. 10). Passion can be defined as “…a strong inclination toward a self-defining activity that one likes (or even loves), finds important, and in which one invests time and energy on a regular basis” (Vallerand, 2012, p. 3). Following that perspective, Vallerand and his colleagues investigated the effects of passion on wellbeing and revealed that being *harmoniously* or *obsessively* passionate could influence wellbeing differently (e.g., Rousseau and Vallerand, 2008; Philippe et al., 2009; Carpentier et al., 2012; Verner-Filion et al., 2017). Additionally, they revealed that positive emotions, flow experiences, ruminations, satisfaction of basic psychological needs, and achievement goals could account for such effects (Rousseau and Vallerand, 2008; Carpentier et al., 2012; Verner-Filion et al., 2017).

Furthermore, De Ridder and Gillebaart (2016) argued that trait self-control represents a key predictor of wellbeing. Trait self-control refers to a dispositional capacity leading to the optimization of the regulation of goal-directed processes, thereby promoting task completion (De Ridder and Gillebaart, 2016). More specifically, because trait self-control “…involves an ‘active self’ that is able to prioritize long-term over short-term goals, even when these short-term goals are immediately gratifying” (De Ridder and Gillebaart, 2016, p. 1), De Ridder and Gillebaart (2016) supposed that trait self-control could enhance wellbeing through initiating (or inhibiting) desired (or undesired) behaviors, then fostering goal attainment and positive emotions. Investigating the effects of trait self-control on wellbeing, authors revealed that positive emotions, promotion focus (i.e., motivational orientation concerned with gains, advancement, and achievement), and prevention focus (i.e., motivational orientation concerned with losses, vigilance, and ought) could account for the effects of trait self-control on happiness (Cheung et al., 2014; Hofmann et al., 2014).

In sum, passion and trait self-control are key to understanding how people can develop high levels of wellbeing. Therefore, the present study aims at examining (a) the relationships between passion, trait self-control, and wellbeing; (b) two models predicting wellbeing; (c) whether trait self-control can mediate the relationship between passion and wellbeing (i.e., model 1); and (d) whether passion can mediate the relationship between trait self-control and wellbeing (i.e., model 2).

### The dualistic model of passion

The dualistic model of passion (Vallerand et al., 2003; Vallerand and Houlfort, 2003; Vallerand, 2008) posits that two dimensions of passion exist: harmonious and obsessive. Harmonious passion results from an *autonomous* internalization of the activity in the self, in such a way that self-initiation, volition, and willingness drive harmoniously passionate individuals' behaviors. With harmonious passion, individuals voluntarily take part in their passionate activity and perceive their activity as being under their control (“My passion for playing football allows me to live a variety of experiences”) and well-integrated with other life domains (“My passion for music is in harmony with other things that are part of me”). Obsessive passion results from a *controlled* internalization of the activity in the self, since internal and external pressure and contingencies drive obsessively passionate individuals' behaviors. With obsessive passion, individuals experience an uncontrollable urge to take part in their activity (“I have the impression that my passion controls me”) and perceive their activity as poorly integrated with other domains of life (“Doing scientific research is so exciting that I sometimes lose controls over it”).

The passionate activity occupies an important space in the identity because it reflects a self-defining activity leading the passionate individual to claim he/she is a practitioner of his/her activity (e.g., “I am a singer!”). Grounded in the self-determination theory (e.g., Deci and Ryan, 2008a,b), the dualistic model of passion posits that harmonious passion originates from autonomous internalization, which “…occurs when people have freely accepted an activity as important for them without any contingencies attached to it” (Vallerand and Miquelon, 2007, p. 252). The dualistic model of passion also posits that obsessive passion originates from controlled internalization, which “…involves internalizing the activity into one's identity because one feels pressured to do so or because some contingencies are attached to the activity, such as feelings of social acceptance or self-esteem” (Vallerand and Miquelon, 2007, p. 252). In sum, Vallerand and his colleagues argue that the more (or the less) the social environment is capable of satisfying individuals' needs for competence, relatedness, and, especially, autonomy, the more autonomous (or controlled) internalization develops for the passionate activity.

In all, while harmonious passion reflects an autonomous regulation, obsessive passion reflects a controlled regulation. For that reason, the dualistic model of passion argues that harmonious (or obsessive) passion is expected to entail adaptive (or maladaptive) health-related outcomes, and many studies have evidenced such assumptions. Indeed, authors showed that harmonious passion predicted positive wellbeing, whereas obsessive passion predicted negative or unpredicted wellbeing (e.g., Vallerand et al., 2007, 2008; Carbonneau et al., 2008; Rousseau and Vallerand, 2008; Philippe et al., 2009; Carpentier et al., 2012; Verner-Filion et al., 2017). More specifically, the authors revealed that harmonious passion has a positive influence on wellbeing through experiencing higher levels of positive emotions, flow (i.e., a state of beatitude and complete concentration on the task), and mastery-approach goals (i.e., the desire to perform the task well; e.g., Rousseau and Vallerand, 2008; Carpentier et al., 2012; Verner-Filion et al., 2017). In contrast, they reported that the detrimental effects of obsessive passion on wellbeing were due to increased and decreased levels of rumination (i.e., repetitive and spontaneous thoughts regarding a specific target) and flow, respectively (Carpentier et al., 2012).

### Can trait self-control mediate the relationship between passion and wellbeing?

Vallerand and colleagues demonstrated the existence of effective affective and cognitive mediators accounting for the relationships between passion and wellbeing. Following such a perspective, the present article aims at examining whether and how harmonious and obsessive passion might influence wellbeing through trait self-control. Trait self-control reflects the stable capability of the self to operate changes in order to adjust the self to the world (Tangney et al., 2004). Specifically, trait self-control promotes facilitative strategies and overrides goal-disruptive impulses (Tangney et al., 2004; Hagger, 2013, 2014). Recent studies have shown that autonomous (or controlled) regulation positively (or negatively) predicted trait self-control (Briki et al., 2015; Briki, 2016), while trait self-control positively predicted positive emotions and wellbeing (e.g., De Ridder et al., 2012; Cheung et al., 2014; Hofmann et al., 2014; Briki et al., 2015; Briki, 2016). In addition, trait self-control appeared to mediate the positive (or negative) effect of autonomous (or controlled) regulation on wellbeing (Briki et al., 2015; Briki, 2016).

Why would autonomous regulation for a specific activity result in greater levels of trait self-control? Briki (2016) addressed that essential question by combining the following views: (a) autonomous self-control depletes low levels of cognitive resources (e.g., Muraven et al., 2007, 2008); (b) autonomous self-control leads to the internalization of values, goals, and behaviors in an autonomous fashion (Deci and Ryan, 1987, 2000); and (c) autonomously internalized goals promote goal selection operations (Carver and Scheier, 1998). Goal selection corresponds to the ability of the self to embrace self-relevant goals and eschew self-irrelevant ones. Based on these literatures, Briki (2016) argued that autonomous regulation would reinforce trait self-control by endorsing (eschewing) goals that are consistent (inconsistent) with the pursued activity. For example, performing physical exercise for autonomous reasons would raise the desire to move toward healthy/desired goals (e.g., eating enough fruit, going to bed early) *and* to move away from unhealthy/undesired goals (i.e., anti-goals; e.g., eating fatty foods, drinking alcohol, smoking, going to bed late). He also argued that controlled regulation would thwart adaptive goal selection operations, thereby leading to endorse inappropriate goals with regard to the pursued activity.

Furthermore, because trait self-control affects long-term goals (Carver and Scheier, 1981; De Ridder and Gillebaart, 2016), and because self-relevant goals are supposed to upgrade in priority (Carver, 2015), one can suggest that autonomous regulation would reinforce trait self-control by setting appropriate long-term/abstract goals related to the pursued activity. Indeed, goals are more than cognitive single-steps, but are rather complex entities that are hierarchically organized in the cognitive system (e.g., Carver and Scheier, 1998; DeYoung, 2015) along temporal (i.e., from long-term to short-term goals; Locke and Latham, 1990) and abstraction-related scales (i.e., from abstract to concrete goals; Carver and Scheier, 1998). For example, being autonomously passionate about physical exercise would raise the desire to move toward long-term/abstract desired exercise goals (e.g., improving one's physical fitness, health, and wellbeing) *and* to move away from long-term/abstract undesired exercise goals (e.g., developing cardiovascular disease, obesity, and depression). Accordingly, autonomous regulation (e.g., harmonious passion) would orientate people's attention toward relevant and suitable long-term/abstract goals (high priority) to the detriment of short-term/concrete goals that would be even immediately gratifying (e.g., eating fatty food) (low priority), whereas controlled regulation would thwart that process. A series of studies support our assumption by revealing that autonomous (or controlled) regulation predicted long-term (or short-term) goals, and that controlled regulation did not predict long-term goals (e.g., Williams and Deci, 1998; Pelletier et al., 2001; see also Deci and Ryan, 2008b for a review).

Finally, we argue here that autonomous regulation (e.g., harmonious passion) would increase trait self-control through cognitive operations of goal selection and reprioritization, thereby promoting the development of self-relevant *and* successful long-term/abstract and short-term/concrete goals, according to the pursued activity. We also expect that controlled regulation (e.g., obsessive passion) would prevent/thwart such a cognitive process. In other words, we presume that commitment to a specific activity (i.e., being passionate for a given activity) may predict a general emotional and cognitive appraisal (i.e., wellbeing) through a stable cognitive ability (i.e., trait self-control).

### Can passion mediate the relationship between trait self-control and wellbeing?

Although we have thus far described the mediating role of trait self-control, we have said little about whether passion can account for the beneficial effect of trait self-control on wellbeing. Hence, in that section, we will propose a model delineating a process from the dispositional level (i.e., trait self-control) to the motivational level (i.e., passion) to self-appraisal (i.e., wellbeing).

Personality traits have been identified as powerful predictors of health-related outcomes (Bogg et al., 2008; Hampson, 2012). According to the neo-socioanalytic theory (e.g., Roberts and Wood, 2006), personality is composed of four distinct personality units—i.e., traits (i.e., stable tendencies to think, feel, and act), motives (i.e., things that people need, desire, and strive to reach), abilities (i.e., acquired skills and knowledge), and narratives (i.e., stories about oneself, others, and one's environment). Each personality unit encompasses a hierarchy of components arranged along an abstraction-based scale—from the most decontextualized to the most contextualized levels—where proximal relations between the components are presumed to be stronger compared to distal relations (i.e., psychological proximity). The theory also posits that strong relations are possible between components pertaining to different personality units. Following that perspective, enjoying exercising (i.e., low-level affect construct) would be psychologically closer to passion for sport and physical activity (i.e., mid-level exercise identity construct) than trait self-control (i.e., top-level conscientiousness construct). Therefore, we suggest that passion may mediate the influence of trait self-control on emotions experienced while performing the activity.

How do such emotions influence wellbeing (i.e., general appraisal)? Diener et al. (2017) suggested that “…affect balance—experiencing more pleasant than unpleasant emotions—is strongly associated with life satisfaction” (p. 91) and that “…frequent but mild positive moods may be sufficient” (p. 94) to developing wellbeing. In other words, the authors indicate that wellbeing results from experiencing repeatedly positive and moderate emotions, supporting studies evidencing that positive emotions develop wellbeing (e.g., Rousseau and Vallerand, 2008; Hofmann et al., 2014). In sum, and in line with authors who demonstrated that trait self-control influenced wellbeing through motivational and emotional constructs (e.g., Cheung et al., 2014; Hofmann et al., 2014), we presume that trait self-control may have a positive impact on wellbeing through passion, which represents a motivational construct deeply rooted in the identity (e.g., Vallerand et al., 2003; Verner-Filion et al., 2017).

### Research overview

The present study aims at examining the predictive factors of wellbeing by building and comparing two distinct models. The first model is arranged from passion to wellbeing, with trait self-control as a midlevel construct. In that model, we posit that passion for a specific activity may foster wellbeing via the mediating effect of trait self-control. The second model is arranged from trait self-control to wellbeing, with passion as an intermediate construct. In that model, we presume that trait self-control may promote wellbeing through experiencing passion for a specific activity. In both models, we assume that trait self-control would promote the setting of facilitative long-term/abstract and short-term/concrete goals, which would lead to promoting goal attainment and, thus, positive emotions. On the basis of the above-mentioned rationale, four categories of hypothesis were proposed:

1. *Relationships between passion and wellbeing:* Consistent with previous studies showing that harmonious passion was positively related to wellbeing, whereas obsessive passion was either negatively related or unrelated to wellbeing (e.g., Carbonneau et al., 2008; Rousseau and Vallerand, 2008; Vallerand et al., 2008; Carpentier et al., 2012), we expect harmonious passion to be positively related to wellbeing. However, no consistent evidence allows us to formulate any prediction regarding the relationship between obsessive passion and wellbeing.
2. *Relationship between trait self-control and wellbeing:* Consistent with previous studies showing that trait self-control was positively associated with wellbeing (e.g., Briki et al., 2015; Briki, 2016), we expect trait self-control to be positively related to wellbeing.
3. *Relationships between trait self-control and passion:* In line with previous studies revealing positive relationships between autonomous functioning and trait self-control, whereas controlled functioning and trait self-control were either negatively related or unrelated (e.g., Briki et al., 2015; Briki, 2016), we expect harmonious passion to be positively related to trait self-control. However, we remained exploratory concerning the relationship between obsessive passion and trait self-control.
4. *Comparison between the two models and mediations:* Given the established theoretical foundations of the two models, we expect them to be effective for predicting wellbeing. Regarding the mediations, and consistent with previous studies indicating that trait self-control mediated the relationship between autonomous functioning and wellbeing (Briki et al., 2015; Briki, 2016), we expect trait self-control to mediate the relationship of harmonious passion with wellbeing (model 1). Moreover, the literature lacks consistency regarding the mediating influence of trait self-control in the relationship between controlled functioning and wellbeing (Briki, 2016; Briki et al., 2015). Thus, the present study remained exploratory concerning that mediation (model 1). In model 2, we expect harmonious passion to mediate the positive effect of trait self-control on wellbeing; however, we formulate no hypothesis regarding the mediating effect of obsessive passion.

## Materials and methods

### Participants

Using an online crowdsourcing platform (ClickWorker^1^), we recruited 509 volunteers from the United States (326 females, 64%, and 183 males, 36%; *M*_age_ = 31.74, *SD*_age_ = 11.05, from 18 to 70 years old; *M*_size_ = 1.63 m, *SD*_size_ = 0.19; *M*_weight_ = 82.09 kg, *SD*_weight_ = 32.11) who reported being passionate about a specific activity (e.g., listening to music, playing video games, reading, photography, biking, cooking, fishing, swimming, blogging). On average, the participants provided a score of 5.94 in passion (*SD* = 0.89). Based on the criterion provided by Philippe et al. (2009) considering that a mean equal to or above “5” (on a 7-point Likert scale) on the passion subscale (see the Section Measures below) would reflect a high level of passion—the present sample of participants might be considered as composed of passionate individuals. Additionally, the participants reported they had been practicing their passionate activity 5.16 times a week (*SD* = 1.81), during 21.30 min per day (*SD* = 61.24), and for 13.17 years (*SD* = 10.22). Lastly, this sample was heterogeneous on several qualitative variables, such as socio-demography (i.e., ethnicity, sex) and medical situation (i.e., chronic mental and physical disease; see Table 1).

**Table 1:** **Socio-demography and medical situation of participants**.

	***n***	**%**
**SOCIO-DEMOGRAPHY**		
**Ethnicity**		
African American	74	14.5
Asian American	33	6.5
Caucasian American	326	64
Hispanic American	43	8.5
Other	33	6.5
**Sex**		
Females	326	64
Males	183	36
**MEDICAL SITUATION**		
**Chronic Physical Disease**		
Yes	55	13.2
No	454	86.8
**Chronic Psychological Disease**		
Yes	67	10.8
No	442	89.2

### Study design and procedure

The present study was performed in line with the recommendations from Qatar University's Institutional Review Board. Before starting the study, the participants read information about the study. Specifically, they were told that the study was designed to examine relationships between passion and feelings, and they read the definition of passion proposed by Vallerand (2012) (see above). The participants were also told that (a) they had to have a passion for an activity to participate in the study, (b) they would respond to questions and their responses would be completely anonymous and confidential^2^, and (c) they would receive a compensation of US$0.45 in exchange for their participation. When the participants accepted to take part in the study, they provided their informed written consent, reported basic information (e.g., socio-demography), and started answering questionnaires. After reporting their passionate activity, the participants completed the following scales, sequentially: (a) harmonious and obsessive passion, (b) general passion, (c) trait self-control, (d) hedonic wellbeing scales, and (e) eudaimonic wellbeing scales.

### Measures

The Passion Scale was composed of three subscales: Harmonious passion (6 items; e.g., “My activity is in harmony with other activities in my life,” α = 0.84), obsessive passion (6 items; e.g., “I have difficulties controlling my urge to do my activity,” α = 0.82), and general passion (4 items; e.g., “This activity is a passion for me,” α = 0.79; Vallerand et al., 2003). The questions were measured on a 7-point Likert scale ranging from “*Not agree at all*” (“1”) to “*Very strongly agree*” (“7”). Trait self-control was measured using the questionnaire developed by Tangney et al. (2004) (13 items; e.g., “I am good at resisting temptation”; α = 0.87; “1” = “*Not at all*,” “7” = “*Very much so*”). Wellbeing combined two concepts: hedonic and eudaimonic wellbeing. To assess hedonic wellbeing, we employed two scales: the Oxford Happiness Questionnaire (Hills and Argyle, 2002; 8 items; e.g., “I am well-satisfied about everything in my life”; α = 0.76; “1” = “*Strongly disagree*,” “6” = “*Strongly agree*”) and the Satisfaction with Life Scale (Diener et al., 1985; 5 items; e.g., “The conditions of my life are excellent”; α = 0.90; “1” = “*Strongly disagree*,” “7” = “*Strongly agree*”). To measure eudaimonic wellbeing, we employed two scales: The Subjective Vitality Scale^3^ (Bostic et al., 2000; 6 items; e.g., “I feel alive and vital”; α = 0.92; “1” = “*Not at all*,” “7” = “*Very true*”) and the Questionnaire of Eudaimonic Well-Being (Waterman et al., 2010; 22 items; e.g., “I can say that I have found my purpose in life”; α = 0.87; “0” = “*Strongly disagree*,” “4” = “*Strongly agree*”).

### Analysis

Because the distributions of all the manifest variables were non-normal^4^ (Shapiro–Wilk test, *p*s ≤ 0.001), we used non-parametric tests: the Partial Least Square Structural Equation Method (PLS-SEM; for assessing the models quality via measurement and structural model analyses; bootstrapping = 1,000 resampling iterations) and Spearman's rho (for correlation analyses between the manifest variables).

### Measurement and structural models

A measurement model gathers several manifest and latent variables. When a latent variable is composed of more than one manifest variable, the quality of the latent variable can be assessed through one of the following benchmarks: (a) the first eigenvalue of the correlation matrix from the principal component analysis is larger than 1.000, whereas the other eigenvalues are lower than 1.000; (b) the internal consistency index (Cronbach's alpha) is higher than 0.700; or (c) the composite reliability index of the latent variables (Dillon-Goldstein's rho) is higher than 0.700 (e.g., Vinzi et al., 2010). Preliminary analyses indicated that “wellbeing” appeared to be an effective latent variable of all wellbeing-related constructs used in this study (i.e., happiness, satisfaction with life, subjective vitality, and eudaimonic wellbeing; see Table 2). The PLS model yielded standardized path coefficients [estimated through ordinary least squares (OLS) regressions], indicating the strength of causal relationships (see **Table 4**).

**Table 2:** **Unidimensionality of wellbeing**.

**Latent Variable Name**	**No. of MVs**	**Cronbach's α**	**D.G.'s ρ**	**PCA Eigenvalues**
Wellbeing	4	0.880	0.918	2.944
				0.487
				0.305
				0.264

*No. of MV, number of manifest variables; D.G.'s ρ, Dillon-Goldstein's rho; PCA, principal component analysis*.

### Assessment of model quality

The goodness-of-fit (GoF) indexes and the coefficient of determination of endogenous latent variables, *R*^2^, assessed the model quality (e.g., Tenenhaus et al., 2005; Henseler et al., 2009). The GoF indexes enabled us to assess the measurement model quality (i.e., outer model GoF index), the structural model quality (i.e., inner model GoF index), or both (i.e., absolute and relative GoF indexes). A GoF index can vary from “0” (i.e., rejection of the model) to “1” (i.e., validation of the model). The critical value for the indexes of relative GoF, outer model GoF, and inner model GoF is 0.900—said differently, if the GoF score is equal to or larger than 0.900—then the model quality is considered as acceptable. Moreover, the critical values for the absolute GoF are 0.010 (small quality), 0.250 (moderate quality), and 0.360 (large quality; e.g., Tenenhaus et al., 2005; Vinzi et al., 2010). Furthermore, *R*^2^ represents a measure of fitness, and when a *R*^2^ was significant the model's quality was acceptable.

### Mediations

A significant mediation can be reported when all of the following conditions are satisfied. Firstly, we must observe a direct and significant relationship between the independent variable and the dependent variable (excluding the interaction of the mediator). Secondly, we must observe significant relationships between the independent variable and the mediator, and between the mediator and the dependent variable. Thirdly, we must observe significant indirect and total effects (including the interaction of the mediator). Fourthly, the size and strength of the mediation, estimated through the value of the variance accounted for (VAF), must be equal to or higher than 20% (Hair et al., 2014; see **Table 5**). More specifically, a VAF score larger than 80% indicates the mediation is full, while a VAF score equal to or larger than 20% and equal to or lower than 80% indicates the mediation is partial. Notwithstanding, when the VAF score is lower than 20%, one should “…conclude that (almost) no mediation takes place” (Hair et al., 2014, p. 225; see **Table 5**).

## Results

### Preliminary analyses

**Figure 1:**
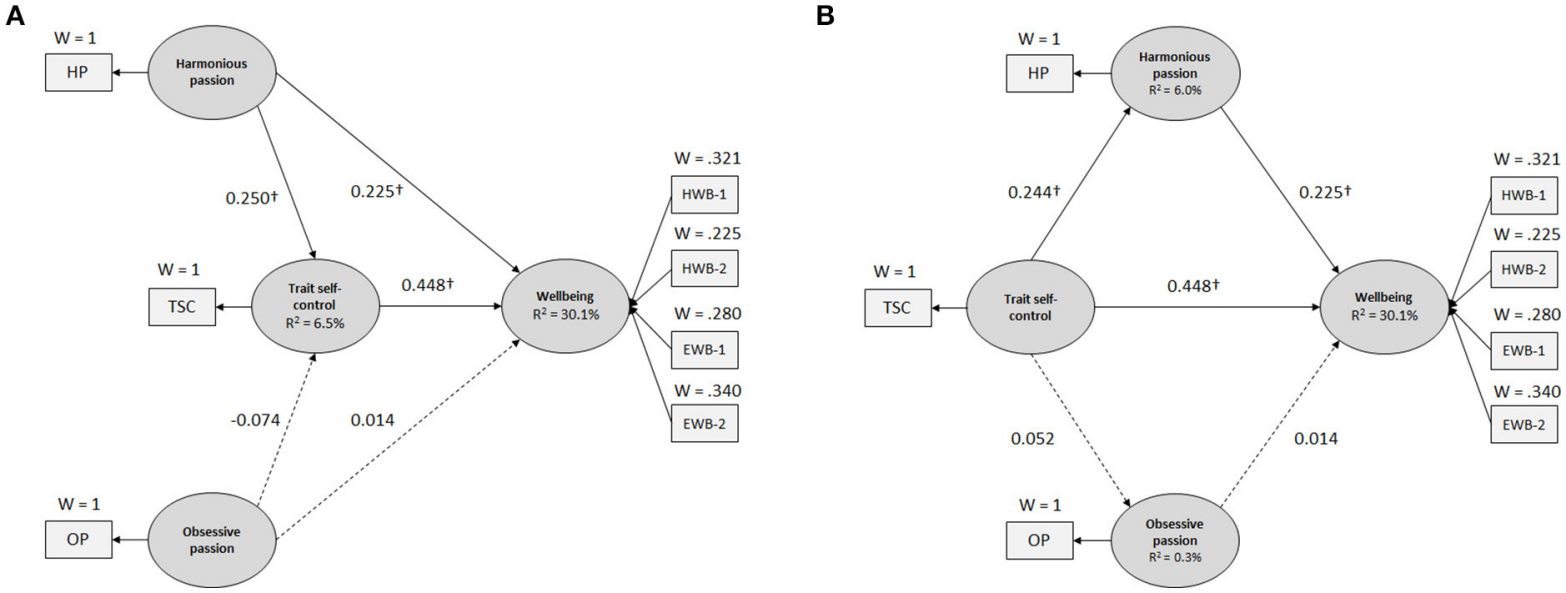
**Structural equation models: (A)** model 1 and **(B)** model 2. All coefficients are standardized and solid lines indicate statistical significance. ^†^*p* < .001 for a two-tailed test. HP, harmonious passion; OP, obsessive passion; TSC, trait self-control; HWB-1, happiness; HWB-2, satisfaction with life; EWB-1, vitality; EWB-2, eudaimonic wellbeing.

The analyses revealed that both models were significant (*model 1:* absolute GoF = 0.366, relative GoF = 0.971, outer model GoF = 0.997, inner model GoF = 0.973, *R*^2^ = 18.300%, *p* < 0.001; *model 2:* absolute GoF = 0.298; relative GoF = 0.980; outer model GoF = 0.997; inner model GoF = 0.982; *R*^2^ = 12.111%, *p* < 0.001; see Figure 1).

### Main analyses

#### Correlations

Correlations revealed positive relationships between harmonious passion, trait self-control, and the four forms of wellbeing (ρs = 0.225 to 0.427, *p*s < 0.001), and no relationships of obsessive passion with the other variables (ρs = −0.044 to 0.008, *p*s > 0.05; see Table 3). The four wellbeing-related constructs were positively associated with each other (ρs = 0.523 to 0.732, *p*s < 0.001; see Table 3).

**Table 3:** **Non-parametric (Spearman's rho) correlations for all manifest variables**.

**Manifest variable**	**1**	**2**	**3**	**4**	**5**	**6**
1. Harmonious passion	–					
2. Obsessive passion	0.072	–				
3. Trait self-control	0.225^†^	−0.044	–			
4. Happiness	0.270^†^	−0.025	0.447^†^	–		
5. Satisfaction with life	0.157^†^	−0.020	0.341^†^	0.683^†^	–	
6. Vitality	0.228^†^	0.008	0.419^†^	0.732^†^	0.684^†^	−
7. Eudaimonic wellbeing	0.427^†^	−0.001	0.401^†^	0.582^†^	0.523^†^	0.636^†^

*The data of all latent variables come from confirmatory factor analysis*.

† *p < 0.001 for a two-tailed test*.

#### Model 1: paths and mediations

The analyses revealed that (a) harmonious passion positively predicted trait self-control (β = 0.225, *p* < 0.001) and wellbeing (β = 0.250, *p* < 0.001), (b) trait self-control positively predicted wellbeing (β = 0.448, *p* < 0.001), and (c) obsessive passion did not predict trait self-control (β = −0.074, *p* > 0.05) and wellbeing (β = 0.014, *p* > 0.05; see Table 4 and Figure 1A). Furthermore, trait self-control appeared to significantly mediate the relationship between harmonious passion and wellbeing (partial mediation: the direct, indirect, and total effects were significant, VAF = 33.136%), but did not mediate the relationship between obsessive passion and wellbeing (the direct, indirect, and total effects were not significant; see Tables 4, 5).

**Table 4:** **Path estimates of the two models**.

**Model**	**Effect**	**Path**	**β**	**SE**	***t*-values**	***p*-values**	***f^2^***
Model 1	Direct (without mediator)	Harmonious passion → Wellbeing	0.359	0.041	8.652	0.000	0.148
		Obsessive passion → Wellbeing	0.055	0.044	1.249	0.212	0.003
	Direct (with mediator)	Harmonious passion → Wellbeing	0.225	0.039	5.842	0.000	0.068
		Obsessive passion → Wellbeing	0.014	0.037	0.366	0.715	0.000
		Trait self-control → Wellbeing	0.448	0.038	11.648	0.000	0.269
		Harmonious passion → Trait self-control	0.250	0.043	5.805	0.000	0.067
		Obsessive passion → Trait self-control	−0.074	0.043	−1.710	0.088	0.006
Model 2	Direct (without mediator)	Trait self-control → Wellbeing	0.500	0.038	13.017	0.000	0.334
	Direct (with mediator)	Trait self-control → Wellbeing	0.448	0.038	11.648	0.000	0.269
		Trait self-control → Harmonious passion	0.244	0.043	5.669	0.000	0.063
		Trait self-control → Obsessive passion	0.052	0.044	1.181	0.238	0.003
		Harmonious passion → Wellbeing	0.225	0.039	5.842	0.000	0.068
		Obsessive passion → Wellbeing	0.014	0.037	0.366	0.715	0.000

**Table 5:** **Mediation analysis for the two models**.

	**Effect**	**Path**	**Mediator**	**Dir. effect (SE) [95% CIs]**	**Indir. effect (SE) [95% CIs]**	**Total effect (SE) [95% CIs]**	**VAF (%)**	**Mediation**
Model 1	Without mediator	HP → WB	N/A	0.359^†^ (0.041) [0.290; 0.427]	N/A	N/A	N/A	
		OP → WB	N/A	0.055 (0.044) [−0.124; 0.139]	N/A	N/A	N/A	
	With mediator	HP → WB	TSC	0.225^†^ (0.039) [0.150; 0.305]	0.112^†^ (0.022) [0.069; 0.155]	0.338^†^ (0.042) [0.258; 0.424]	33.136	Partial
		OP → WB	TSC	0.014 (0.037) [−0.062; 0.086]	0.033 (0.020) [−0.075; 0.006]	−0.019 (0.044) [−0.111; 0.064]	N/A	
Model 2	Without mediator	TSC → WB	N/A	0.500^†^ (0.038) [0.427; 0.570]	N/A	N/A	N/A	
	With mediator	TSC → WB	HP	0.244^†^ (0.043) [0.162; 0.324]	0.056^†^ (0.014) [0.032; 0.085]	0.503^†^ (0.035) [0.433; 0.570]	11.144	No
		TSC → WB	OP	0.502^†^ (0.039) [0.428; 0.562]	0.002 (0.003) [−0.005; 0.009]	0.033 (0.040) [−0.048; 0.113]	N/A	

† *p < 0.001 for a two-tailed test. VAF > 80% = Full mediation, 20% ≤ VAF ≤ 80% = Partial mediation, and VAF < 20% = No mediation*.

*HP, harmonious passion; OP, obsessive passion; TSC, trait self-control; WB, wellbeing; VAF, variance accounted for; N/A, not applicable*.

#### Model 2: paths and mediations

The analyses revealed that (a) trait self-control positively predicted harmonious passion (β = 0.224, *p* < 0.001) and wellbeing (β = 0.448, *p* < 0.001), (b) trait self-control did not predict obsessive passion (β = 0.052, *p* > 0.05), and (c) harmonious passion positively predicted wellbeing (β = 0.225, *p* < 0.001), whereas obsessive passion did not predict wellbeing (β = 0.014, *p* > 0.05; see Table 4 and Figure 1B). The mediating effect of harmonious passion appeared to be significant (the indirect effect was significant, *p* < 0.001), but the VAF value was lower than 20% (VAF = 11.144%; see Tables 4, 5). No mediating effect of obsessive passion was found (see Tables 4, 5).

## Discussion

Using different frameworks, such as the dualistic model of passion and the neo-socioanalytic theory, the present study sought to examine the interrelationships between passion, trait self-control, and wellbeing, as well as two models designed to predict wellbeing. In model 1, we examined the relationships between passion and wellbeing, with trait self-control as an intermediate construct. In model 2, we proposed that the dispositional construct (i.e., trait self-control) could predict wellbeing, with passion for an activity as a midlevel construct. Then, we examined whether trait self-control could mediate the effect of passion on wellbeing (model 1), and whether passion could be considered as another mediator of interest for accounting for the relationship between trait self-control and wellbeing (model 2).

### Relationships between passion, trait self-control, and wellbeing

The results revealed that harmonious passion was positively related to wellbeing, whereas obsessive passion was unrelated to that variable (see Table 3). This supports the view that harmonious passion can promote wellbeing (e.g., Vallerand, 2012, 2015), as well as previous studies showing that harmonious passion was positively related to wellbeing, whereas obsessive passion was either negatively related or unrelated to wellbeing (e.g., Vallerand et al., 2007, 2008; Carpentier et al., 2012). According to the dualistic model of passion, harmoniously passionate people would be more likely to report high levels of wellbeing compared to obsessively passionate people, because harmonious (obsessive) passion results from an autonomous (controlled) internalization of the passionate activity into the self, resulting from the satisfaction (dissatisfaction) of psychological innate needs. When harmonious passion is developed, people would experience a sense of control over their activity and willingly embrace other activities, resulting in the experience of positive emotions and wellbeing. Additionally, such a functioning may lead to peak experiences, such as flow (Carpentier et al., 2012). Following this perspective, one might suggest that harmonious passion might also precipitate experiences of psychological momentum (i.e., states of energization and impetus initiating a trajectory of performance outcomes; Iso-Ahola and Dotson, 2016; Briki, 2017).

The results also revealed that trait self-control was positively related to wellbeing (see Table 3), supporting previous studies displaying positive associations between trait self-control and wellbeing (e.g., Cheung et al., 2014; Briki et al., 2015; Briki, 2016). The authors proposed that trait self-control would promote wellbeing by fostering effective strategies and positive emotions (Cheung et al., 2014; De Ridder and Gillebaart, 2016), and such emotions would result from the capability of trait self-control to manage conflicting desires, facilitate task completion, and foster success experiences (Hofmann et al., 2012, 2014; Hagger, 2013, 2014; De Ridder and Gillebaart, 2016). The results also revealed that trait self-control was positively related (or unrelated) to harmonious (or obsessive) passion (see Table 3). These results support those of previous studies that reported (a) positive associations between trait self-control and autonomous functioning (Briki et al., 2015; Briki, 2016) and (b) no association between trait self-control and controlled functioning (Briki et al., 2015).

### Comparing the two models predicting wellbeing

In model 1, actively practicing one's passionate activity for autonomous reasons (i.e., harmonious passion) represented a context whereby trait self-control could grow, and such a growth was supposed to be responsible for wellbeing enhancement. Interestingly, this model echoes the *social investment principle*, which posits that committing to social institutions (e.g., work, marriage, family) induces personality development because endorsing valued social roles (e.g., a university professor) would lead to adopting specific goals (e.g., obtaining a promotion), incorporating specific norms and expectations (e.g., dominance, agreeableness, conscientiousness, abnegation), and modifying one's identities (Roberts and Wood, 2006). Grounded in the neo-socioanalytic approach (e.g., Bogg et al., 2008), model 2 proposed an organization arranging the different variables from a top-level construct of the self (i.e., trait self-control) to a low-level affect construct (i.e., positive emotions and feelings), with a mid-level exercise identity construct (i.e., passion for an activity). The results indicated that models 1 and 2 were both significant, thus supporting our predictions and validating the two approaches used in the present study.

In model 1, the results showed that harmonious passion positively predicted trait self-control and wellbeing (see Table 4 and Figure 1A). This result can be explained by the fact that self-relevant activities would promote goal selection and reprioritization processes (Carver and Scheier, 1998; Carver, 2015), thus echoing Carpentier et al.'s (2012) study showing that harmonious passion promoted adaptive cognitive responses (e.g., deep state of concentration in the task). Moreover, the mediation analyses revealed that trait self-control partially mediated the relationship between harmonious passion and wellbeing (see Table 5), supporting previous studies showing that trait self-control partially mediated the positive influence of autonomous motivation on wellbeing (Briki et al., 2015; Briki, 2016). Hence, our result enriches the predictions of the dualistic model of passion by suggesting that harmonious passion can foster agreeable affective experiences and wellbeing via increasing the effectiveness of self-regulatory processes.

The results also showed that trait self-control did not mediate the relationship between obsessive passion and wellbeing (see Table 5), and obsessive passion neither predicted trait self-control nor wellbeing. One can explain these results by the fact that controlled regulation driving obsessive passion would prevent the activation of facilitative cognitive processes (e.g., long-term goal selection), which are well-known to help make progress toward desired goals and experience positive emotions. The dualistic model of passion indicates that obsessive passion makes people suffer from living under the control of their favorite activities, preventing them from considering and using time-outs as fruitful opportunities to energize themselves and have fun (e.g., performing a physical exercise session with one's children).

In model 2, the results revealed that obsessive passion neither predicted trait self-control and wellbeing nor mediated the positive effect of trait self-control on wellbeing, supporting the view that obsessive passion represents a maladaptive (or, at least, a non-adaptive) construct. In contrast, the results indicated that harmonious passion mediated the relationship between trait self-control and wellbeing (the indirect effect was significant); however, the size of the mediating effect indicated that (almost) no mediation would take place (VAF = 11.144%). Therefore, one can suggest that the beneficial effect of trait self-control on wellbeing would be essentially due to the intervention of self-regulatory mechanisms (De Ridder and Gillebaart, 2016), but that harmonious passion *may* also account for such a relationship. Indeed, this result incites the pursuit of an investigation regarding the potential mediating effect of harmonious passion. Finally, our results support DeYoung's (2015) view “…that the highest and most enduring levels of well-being should be achieved when one's characteristic adaptations are not only well-adapted to one's particular life circumstances, but also well-integrated—that is, minimally conflicting with each other, with one's traits, and with innate needs” (p. 53).

## Conclusion and perspectives

The present study is the first to examine the relationships between passion, trait self-control, and wellbeing, and supports the view that trait self-control and harmonious passion represent not only adaptive and powerful constructs, but also key determinants of wellbeing (e.g., Tangney et al., 2004; Bogg, 2016; Verner-Filion et al., 2017). However, this study is not without limitations. Firstly, it was correlational; hence, future investigations should employ stronger (experimental) causal designs. We suggest that future research might be directed toward examining the effects of intervention programs designed to promote harmonious passion on trait self-control and wellbeing. Secondly, as for any self-report measure-based investigations, social desirability biases might have produced contamination of affective responses, resulting from cognitive reasoning and judgments. Nonetheless, by informing the participants about the anonymous and confidential nature of this study, the methodology of this investigation attempted to limit such biases.

This study also brought evidence that the two models were effective for predicting wellbeing. As a result, different studies could take place within each of the models. Following the perspective of model 1, further studies should examine the effects of autonomy-supportive (vs. controlling) contexts or, more generally, the effects of autonomy/controlling-related cues on the development of trait self-control and wellbeing over several weeks. In line with the perspective proposed by model 2, one could examine whether people who have more trait self-control would develop higher levels of harmonious passion and wellbeing. Moreover, combining both perspectives, we believe that time-based studies exploring the interdependence between personality and health may contribute to a better understanding of wellbeing (Bogg, 2016). Indeed, if we consider that passion, trait self-control and wellbeing may fluctuate over time and display non-linear relationships, then employing a longitudinal approach could help examine the reciprocal effects between harmonious passion and trait self-control for predicting wellbeing. Further studies should measure the needs satisfaction underlying the development of passion (Verner-Filion et al., 2017), since research has found that autonomous functioning could be shaped differently according to cultural and societal characteristics, such as self-construal (e.g., independent and interdependent feature) and social rank (Walker et al., 2011; Walker, 2016). Following such a perspective, future studies should examine the links between needs satisfaction, harmonious passion, trait self-control, and wellbeing.

From an applied standpoint, this study invites to tackle obesity and cardiovascular diseases in response to physical inactivity by promoting harmonious passion for exercise among the general population. Parents, teachers, managers, coaches, and psychologists should pay attention to people's perceptions of autonomy, relatedness, and competence. Considering people as valuable human beings (and not as means to attaining goals), offering them the opportunity to choose, involving them in the decision-making process, truly listening to and supporting them while facing challenges and difficulties, offering the chance to acquire new skills, etc., might develop these perceptions. We speculate that repeating such supportive behaviors over time should contribute to promoting a positive internalization of the given activity (e.g., work, physical exercise) into the self, thereby promoting harmonious passion, trait self-control, and wellbeing.

## Ethics statement

The study design was exempt from Qatar University's Institutional Review Board. The author of the article successfully completed the National Institutes of Health Web-based training course “Protecting Human Research Participants.”

## Author contributions

WB conceived the study, collected and analyzed the data, and wrote and revised the article.

### Conflict of interest statement

The author declares that the research was conducted in the absence of any commercial or financial relationships that could be construed as a potential conflict of interest.

## Footnotes

^1^http://www.clickworker.com

^2^The researcher had no contact with the participants and had not received any personal information about them from the team of the crowdsourcing online platform.

^3^The Subjective Vitality Scale refers to a revised version of the vitality scale developed by Ryan and Frederick (1997).

^4^This supports Astrachan et al.'s (2014) view regarding the non-normality of distributions in social sciences, and this explains why we chose PLS-SEM (XLSTAT-PLS, Addinsoft, version 2016.06.37018) over CB-SEM (e.g., AMOS) for computing our SEM analyses.
